# P165: Perception of health care employee related to handwashing practice and insertion of the patient in this context

**DOI:** 10.1186/2047-2994-2-S1-P165

**Published:** 2013-06-20

**Authors:** MM Baraldi, C Santoro, C Scmitt, M Simoneti, C Lovatto

**Affiliations:** 1 Scih, Alemão Oswaldo Cruz Hospital, São Paulo, Brazil; 2 Uti, Alemão Oswaldo Cruz Hospital, São Paulo, Brazil

## Introduction

Considering that handwashing responsabilityis mainly on health area professionals, the survey of employees' perception helps to orientate the implementation of educational measures in the promotion of improvements and and can also offer subsidies to stimulate patients' active participation in the search for health.

## Objectives

Evaluate the health care employees perception regarding the best practices of handwashing, including the patient involvement in the shared responsability of this handwashing practice

## Methods

It is about a descriptive, quantitative study performed at a midsize, private hospital located in São Paulo, Brazil.

Among the actions performed in the Annual Handwashing Campaign in 2012, pamphlets with closed questions were distributed, allowing the evaluation of 363 healthcare employees.

The variables for evaluation were considered: the importance of the 5 moments practice, the performance of handwashing opportunities, the importance given to the patient involvement, handwashing as part of care and the quality of alcohol gel.

## Results

The analysis of collected data has showed that 99% of employees consider of “much importance” the 5 Moments practice, 58% consider fulfill 100% of opportunities. When evaluated the importance given to the patient involvement in the improvement process, 93% of the employees consider it very important, 3% indifferent and 4% refer moderate importance. 100% of the employees consider that handwashing is part of the health process. 81% of these consider that the family and patient proactivity help increase the implementation of handwashing measures, while 19% of the employees do not agree. The employees evaluated the quality of alcohol gel as: 61% very good and 38% good.

## Conclusion

The results found show a homogeneous awareness on the practice of handwashing and also that the perception of the importance of the patient participation in the shared responsability already is a fact, which allows the implementation of new multiprofessional strategies, stimulating the patient to take an active part in his own care.

## Disclosure of interest

None declared

